# The Use of Animal’s Body, Scrotal Temperature and Motion Monitoring in Evaluating Boar Semen Production Capacity

**DOI:** 10.3390/ani12070829

**Published:** 2022-03-24

**Authors:** Vasiliki Stravogianni, Theodoros Samaras, Constantin M. Boscos, John Markakis, Evdokia Krystallidou, Athina Basioura, Ioannis A. Tsakmakidis

**Affiliations:** 1 School of Veterinary Medicine, Faculty of Health Sciences, Aristotle University of Thessaloniki, 54627 Thessaloniki, Greece; vikistrab@gmail.com (V.S.); pboscos@vet.auth.gr (C.M.B.); 2 School of Physics, Faculty of Sciences, Aristotle University of Thessaloniki, 54124 Thessaloniki, Greece; theosama@auth.gr (T.S.); jmarkakis@gmail.com (J.M.); 3 American Farm School, Marinou Antipa 54, P.O. Box 23, 55102 Thessaloniki, Greece; ekryst@afs.edu.gr; 4 Department of Agriculture, School of Agricultural Sciences, University of Western Macedonia, 53100 Florina, Greece; abasioura@uowm.gr

**Keywords:** sensor signals, thermal camera, boar semen, fertility, pig, biomedicine

## Abstract

**Simple Summary:**

The evaluation of male animals’ fertilizing capacity is based on laboratory-performed tests, as well as on the in vivo and in vitro reproductive outcome. Boar fertility is strongly associated with a pig farm’s financial status and income. The scientific community attempts to increase the predictive reliability of male fertility to ensure the profitability of the pig industry. In this direction, a different approach, using biomedical thermal and motion sensors, is utilized. This study investigates the correlation of collected data by biomedical sensors with variables well known as their predictable value semen quality. Strong correlations are revealed, encouraging the further application of biomedical methods in the assessment of boars’ fertilizing ability.

**Abstract:**

Biomedical measurements by specialized technological equipment have been used in farm animals to collect information about nutrition, behavior and welfare. This study investigates the relation of semen quality (CASA analysis, viability, morphology, membrane biochemical activity and DNA fragmentation) with boar behavior during ejaculation. Sensors were placed on the boar’s body. Movement features were collected using an inertial measurement unit (IMU), comprising an accelerometer, a gyroscope and a magnetometer. Boar, scrotal and dummy temperatures were measured by an infrared (IR) camera and an IR thermometer, while the face salivation of the boar was recorded by a moisture meter (also based on IR technology). All signals and images were logged on a mobile device (smartphone or tablet) using a Bluetooth connection and then transferred wirelessly to the cloud. The data files were then processed using scripts in MATLAB 2021a (MathWorks, Natick, Massachusetts) to derive the necessary indices. Ninety-four ejaculates from five boars were analyzed in this study. The statistical analysis was performed in the Statistics and Machine Learning Toolbox of MATLAB 2021a using a linear mixed effects model. Significant and strong negative correlations (*R*^2^ > 0.5, *p* ≤ 0.05) were observed between boar, dummy and scrotal temperature with the progressive, rapid and slow movement of spermatozoa, VCL (curvilinear velocity), VSL (straight line velocity) and ALH (amplitude of lateral head displacement) kinematics. The volume of the ejaculate was correlated with the scrotal and dummy temperature. Dummy’s temperature was negatively correlated with BCF (beat/cross-frequency), viability and total time of ejaculation, while it was positively correlated with abnormal morphology. Body temperature was negatively correlated with BCF. Positive correlations were noticed between VAP (average path velocity) and total time of ejaculation with body acceleration features, as well as between the overall dynamic body acceleration (ODBA) and total time of ejaculation. In conclusion, the use of biomedical sensors can support the evaluation of boar sperm production capacity, providing valuable information about semen quality.

## 1. Introduction

Boars’ fertilizing capacity affects productivity, being a key factor for the development, competitiveness and performance of pig farming. The worldwide application of artificial insemination (AI) maximizes the need for high-quality boar semen. However, boar ejaculate demonstrates quality variations during the year because of animal and nonanimal origin factors. Among them, boar age, health and stress status, season and environmental conditions and the frequency of semen collection significantly influence boar semen quality [1,2]. It is notable that boar semen composition shows 25–30% changes during the year period [3].

Semen collection processing is characterized by a sequence of boar movements to reach and mount a dummy, to present erection, to exhibit face saliva and to begin and complete the ejaculation. According to pig farm experiences, faster access to the dummy, faster appearance of a full erection and faster completion of the ejaculation process usually characterize the best males. Levis and Reicks, 2005 [4] reported that boars which ejaculated faster provide semen with a higher number of spermatozoa, while boar saliva pheromones stimulate the females contributing to the onset of estrus, as well as to the first successful ovulation of the gilts [5]. The above-mentioned studies reinforce the opinion that boar libido and behavior affect the collected semen quality, but no recent, in-depth and well-documented research exists that could confirm this. 

A concerning semen evaluation and safe and early prognosis of sub- or infertile boars can be achieved combining the results of multiple and specialized laboratory sperm diagnostic tests [6]. Recently, modernized and automated real-time data recording systems have been used to monitor farm animals’ behavior, movement, physiology, nutrition and welfare [7,8]. Thermal cameras and sensors are placed on or around the animal’s body to record vital parameters. The use of this equipment enables the recording of animal behavior in a less invasive manner. The obtained data of signals and images can be converted and processed by specialized software. 

Animal body and scrotal temperatures, which affect spermatogenesis, can be measured by infrared cameras. Infrared thermography detects the emitted infrared radiation, depending on body temperature. Animal stress (thermal, sonic, psycho-kinetic, etc.) affects both libido and sperm quality [9]. In pigs, sensors and thermal cameras have been used to mainly record their activity, leading to an accurate diagnosis of laminitis [10,11], the prediction of farrowing [12,13,14] and estrus onset time [15]. 

During ejaculation, the real-time recording of boar behavior, movement, body and scrotal temperature, salivation, total processing time, and the correlation or these measurements with the qualitative sperm characteristics could potentially create a prognostic model of boar fertilizing capacity. To our knowledge, there exist no reports investigating the relation of boar sperm characteristics with data recorded by biomedical techniques and reflecting the animal’s behavior during the ejaculation process. All the relative previous research focused on sows. Therefore, the primary objective of this study is to demonstrate the feasibility of recording and evaluating boar behavior during the process of ejaculation by biomedical sensors. Another objective is to correlate the collected biomedical information expressed in the form of specific indices with the semen tested qualitative characteristics; thus, supporting the usefulness of boars’ monitoring by biomedical techniques, as well as their potential use as an additional reliable tool for the evaluation of boar ability to produce high-quality semen.

## 2. Materials and Methods

The study was approved by the Ethics Committee on Animal Use of the Aristotle University of Thessaloniki, Greece, and all operations were carried out according to the university’s guidelines for animal research (96385-19929/2020-Project Number: HFRI-FM17-2040). All reagents and chemicals used in this study, were purchased from Sigma-Aldrich (Seelze, Germany) unless otherwise specified. 

### 2.1. Animal Management, Semen Collection and Experimental Design

The experiments were carried out on a commercial Greek pig farm, with a capacity of 150 sows. Five (5) adults (12–20 months old), healthy boars (three Pietrain, one Landrace breed and one crossbred Pietrain × Duroc), active in the AI program of the farm, were involved in this study. Boars were housed in individual pens (8 m^2^) in a chamber under controlled conditions of temperature range of 15–25 °C, humidity and ventilation. They were fed according to internationally accepted data for balanced diet and ad libitum water access [16]. Over the span of one year, 94 ejaculates (18–19 ejaculates/boar) were collected and examined for the study’s purposes. Each examined ejaculate was obtained bi-weekly using the gloved hand technique, while the gelatinous portion was discarded. The interval between two consecutive ejaculations of each boar was 2–3 days, because they were also active for the routine insemination program of the farm. Each ejaculate was transported to the farm’s laboratory, where it was initially evaluated and extended by MR-A@ Extender (KUBUS, Madrid, Spain) to final concentration 30 × 10^6^ spermatozoa/mL. The extended semen was re-examined microscopically, and semen samples with total motility ≥70%, viability ≥75% and morphological abnormalities ≤20% were packaged in vials of insemination (100 mL), used for AI in farm, while one semen dose was transported (17 °C) in 60 min to the Unit of Biotechnology of Reproduction, Clinic of Farm Animals, Faculty of Veterinary Medicine, Aristotle University of Thessaloniki, for further analysis.

### 2.2. Biomedical Equipment and Measurements

#### 2.2.1. Equipment

A chronometer was used to record the duration of the semen collection process, which was established as the spent time from the entrance of the boar to the semen collection pen up to the completion of the ejaculation process. Sensors were placed on boar’s body. An inertial measurement unit (IMU) (MBIENTLAB, San Francisco, CA, USA) comprising an accelerometer, a gyroscope and a magnetometer to collect movement features was placed by a special collar on the nape of the boar. Triaxial acceleration data were recorded with a sampling rate of 100 Hz. It was assumed that the standard deviation of the measured acceleration during the collection process would make a good proxy for the vigor of the boar’s motion. The overall dynamic body acceleration (ODBA) was also calculated with a moving average window of 20 s using a script in MATLAB 2021a (MathWorks, Natick, MA, USA). Animal’s body, scrotal, and dummy temperatures were measured by an infrared (IR) camera (FLIR ONE Pro, FLIR Systems, Wilsonville, OR, USA) and an IR digital thermometer, while the face moisture of the boar was recorded by a moisture meter (FLIR MR160, FLIR Systems, Wilsonville, OR, USA). All signals and images were logged on a mobile device (smartphone or tablet) using a Bluetooth connection and then transferred wirelessly to the cloud. 

#### 2.2.2. Data Collection and Processing

**Figure 1:**
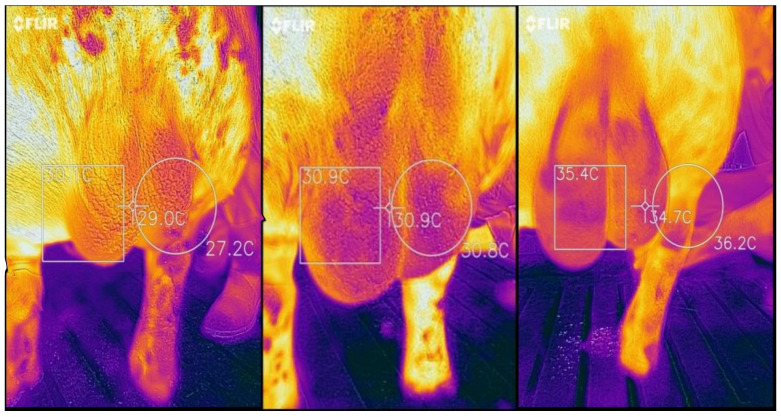
Thermal image of three boar scrotum’s surface areas (square, cross and circle area) during ejaculation.

All measurements were taken from the time of boar entering the sperm collection pen up to the completion of the ejaculation. Concerning the scrotal temperature, pictures were taken every 30 s. Each thermal image included three areas of the scrotum surface (square, cross and circle areas) to measure temperature over as large an area as possible and with greater accuracy (Figure 1). A MATLAB script was used to calculate the average of square, cross and circle areas of scrotum from each picture and, eventually, a median value from all pictures was taken during the sperm collection process.

The boar body and dummy temperatures were measured by an infrared thermometer, just before the animal’s final attempt to mount on the dummy. The temperature of the dummy was measured in the central–middle point of its’ upper surface. 

**Figure 2:**
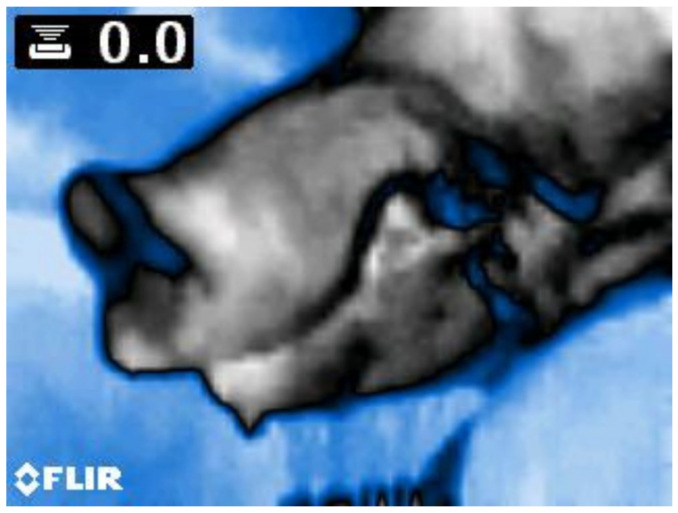
Boar salivation and face moisture imaging during ejaculation.

Concerning face moisture, pictures were taken every 30 s during ejaculation by the moisture meter (Figure 2). Image processing with a MATLAB script could identify the number of “wet pixels” in the picture and result in a median value for all pictures recorded during the sperm collection process.

### 2.3. Semen Analysis

#### 2.3.1. Computer-Assisted Semen Analysis (CASA Analysis)

Computer-assisted semen analysis (CASA-Sperm Class Analyser^®^, Microptic S.L., Automatic Diagnostic Systems, Barcelona, Spain) was applied to measure the following kinematics: (i) total motility %; (ii) progressive spermatozoa %; (iii) rapid, medium and slow spermatozoa % (10 < slow < 45 < medium < 75 < rapid m/s); (iv) VCL—curvilinear velocity (μm/s); (v) VSL—straight line velocity (μm/s); (vi) VAP—average path velocity (μm/s); (vii) ALH—amplitude of lateral head displacement (μm); (viii) BCF—beat/cross-frequency (Hz); (ix) LIN—linearity (VSL/VCL × 100); (x) STR—straightness (VSL/VAP × 100); (xi) WOB—wobble (VAP/VCL × 100). Software configuration and final analysis were performed as reported by Michos et al. [17].

#### 2.3.2. Assessment of Viability and Morphology

Viability and morphology were evaluated after eosin-nigrosine semen double staining [18]. Two hundred sperm cells were estimated per slide (×1000) to provide the rate of live/dead and morphological normal or abnormal sperm cells.

#### 2.3.3. Assessment of Sperm Membrane Biochemical Activity

Hypo-osmotic swelling test (HOST) was applied according to Michos et al. [17], performing a minor alteration of Vazquez et al.’ [19] protocol.

#### 2.3.4. Assessment of Sperm DNA Fragmentation

The acridine orange test (AOT) was applied to determine the sperm DNA integrity [20].

### 2.4. Statistical Analysis

The statistical analysis was performed in MATLAB 2021a (Statistics and Machine Learning Toolbox) using linear mixed-effects models. For each model, one variable was chosen as the predictor variable and another variable as the response variable, assuming a fixed effect between them. The random-effect terms for intercept and predictor variables were considered uncorrelated. The grouping variable was always the boar. The coefficients of determination (*R*^2^) and linear regression, as well as the 95% confidence interval (CI) for the latter, were derived.

## 3. Results

The correlation results between sperm variables, duration of ejaculation and thermal collected data are presented in Table 1.

Significant and strong negative correlations (*R*^2^ > 0.5, *p* ≤ 0.05) were revealed between dummy, scrotal and boar body temperatures with many sperm characteristics, such as the progressive and rapid movement of spermatozoa, VCL, VSL, ALH, and volume of the ejaculation. Moreover, dummy temperature was negatively correlated with BCF, viability and total time of ejaculation process, while body temperature was negatively correlated with BCF. Positive correlations were observed between all measured temperatures and spermatozoa with slow movement, while the dummy temperature was also positively correlated with the morphological abnormalities of spermatozoa.

The analysis of collected data revealed significant and strong correlations between sperm variables and sensor signals (*R*^2^ > 0.5, *p* ≤ 0.05). These results are presented in Table 2.

Positive correlations were noticed between the total time of sperm collection with ODBA and acceleration, as well for VAP with acceleration. Furthermore, significant and strong positive correlations were demonstrated after the analysis of the collected data between salivation, sensor and thermal measurements (*R*^2^ > 0.5, *p* ≤ 0.05). These results are presented in Table 3.

Positive correlations were observed between all measured temperatures, as in further between the acceleration and salivation with ODBA. 

No significant and strong correlations were obtained between thermal and sensor variables and the semen characteristics of HOST-positive spermatozoa and DNA fragmentation.

## 4. Discussion

The prognosis of male fertility is an important issue for domestic livestock worldwide. The assessment of boar sperm fertilizing ability is required to support a successful AI, which reflects a pig farm’s profitability. However, standard methods for the accurate prediction of male fertility are not yet available. The combination of different laboratory semen evaluation tests makes the prediction of male infertility more reliable [6]. To achieve more accurate prognoses, various scientific technologies such as biomedicine, proteomics, genomics and biochemistry have been employed, clarifying sperm-specific processes.

In this study, less invasive techniques were used to evaluate boar behavior and activity under the sperm collection process. A possible behavior variability among the boar genetic lines was not an objective of this study, while the number of available boars in this farm could not support this kind of research. Sensors were placed on or around the boar’s body; face salivation, scrotal, body and dummy temperatures were measured and correlated with sperm quality traits of well-known association with fertility. Normal spermatogenesis depended on the normal thermoregulation of the scrotum. Mammalian teste temperature is approximately 4–5 °C lower than the body’s temperature [9]. Any fever disease increases boar body and scrotal temperature, while the dummy temperature, which was also measured in this study, is affected by and serves as a good proxy for the environmental temperature of the pig farm facilities. Negative effects of the environmental heat stress on boar sperm motility and morphology were revealed many years ago [21]. According to [22], the increase in scrotal temperature led to hypoxia, increasing the reactive oxygen species (ROS) production. This process induces hyperoxidation affecting sperm membranes’ integrity and fluidity, as well as major steps of the fertilization mechanism, such as the capacitation and sperm–zona pellucida interaction. The sensitivity of spermatozoa to heat stress is reflected in the decrease in sperm quality based on DNA fragmentation, kinematics, morphology, viability, membrane biochemical activity and mitochondrial membrane potential [17,23]. In the present study, the measured temperatures were not correlated with sperm DNA fragmentation and HOST-positive spermatozoa, although it would be desirable since these two parameters are of high importance for the prognosis of pig fertility. Michos et al. [17] reported a significant relationship of HOST outcome with the percentage of live-born piglets, while Yeste et al. [24] related HOST results with the expected litter sizes, supporting this assay’s prognostic value. Moreover, it is well known that a high rate of DNA fragmentation reflects a low reproductive performance. Roca et al. [25] reported that low litter sizes arise from DNA damage of >20%. In our study, no ejaculates with significant percentages of DNA fragmentation were found. In fact, a few ejaculates of DNA fragmentation lower than 0.3% were revealed. This result did not reflect a high possibility of field fertility decrease. Moreover, considering that boars are usually examined and sorted before their export to pig farms, the chance of finding an ejaculate with high sperm nuclear DNA fragmentation between the five used boars was low. Concerning HOST, it seems that the changes of other sperm parameters were stronger depending on temperature alterations compared to sperm membrane biochemical activity.

On the other hand, strong negative correlations were obtained between the measured temperatures and major semen variables, such as progressive motility, rapid movement spermatozoa and VCL, VSL, ALH and BCF kinematics. The fertilization process is a chain of events, where the chance of a single spermatozoon to reach the oviduct and fertilize the oocyte is dependent on the combined transport mechanism via the uterus muscle contractions and good sperm motility, as well as on molecular level changes. With respect to the boar fertilizing capacity prognosis, progressive motility is the most routinely evaluated and trustable variable. A previous study reported that a motility of <60% negatively affects in vivo and in vitro fertility parameters such as litter size, farrowing rate and penetration rate, respectively [26]. In addition, other studies documented a high relation of progressive motility with boar field fertility based on the farrowing rate and total born piglets [27,28,29]. Regarding the kinematic variables which strongly correlated with the temperature values of the present study, it must be noted that all of them affect fertility. Holt et al. [30] revealed a positive correlation of litter size with VCL, VSL, ALH and BCF. The VCL and BCF variables have been positively related to the farrowing rate [27]. Furthermore, VSL and ALH variables have been negatively correlated to litter size [27]. Moreover, a positive correlation between BCF and the number of stillborn piglets has been previously reported [31]. Strong correlations were not revealed between the measured temperatures and LIN, STR and VAP variables. It seems that the weak correlation of VAP covered the effect of the strong correlation of VSL, leading to a weak correlation of STR (VSL/VAP), while a similar correlation of VSL and VCL was not expressed as their mathematical quotient (LIN = VSL/VCL). Independent of the positive or negative effect of sperm kinematics on fertility outcome, a continuous measurement of body, scrotal and dummy temperature during the ejaculation process can provide useful prognostic information about semen quality and fertility. 

In the present study, the dummy measured temperature was negatively correlated with ejaculate’s volume and viability, but it was positively correlated with morphological abnormalities, while the scrotal temperature was negatively correlated with ejaculate’s volume. The concentration, volume and viability are the three most major interdependent parameters with the number calculation and preparation of AI semen doses. Viability expresses the percentage of spermatozoa with intact membranes, but not the sperm functionality. Many authors have not found a correlation between this parameter and pig farm reproductive performance factors, such as the farrowing rate, litter size and live-born piglets [17,31,32], while some others reported a strong correlation with the nonreturn to the estrus rate and litter size [33]. However, viability is a strict criterion for accepting or rejecting an ejaculation for further processing and use in AI. The low volume of the ejaculate excludes its further processing, while a high volume of it in terms of normal concentration values ensures the production of a high number of semen doses supporting the income of AI centers. Concerning the morphological abnormalities, a rate lower than 25% is needed to approve a boar ejaculate. Abnormal spermatozoa affect normal movement and, in some cases, their fecundation ability. Negative correlations between boar sperm abnormalities, the farrowing rate and litter size were observed in previous studies [34,35], while the combination of sperm morphology and DNA fragmentation traits revealed a predictable value of the farrowing rate [6]. Considering the results of the present study, a significant finding was obtained, indicating that thermal boar monitoring during semen collection processing at least provides valuable information about an initial decision to deny or accept an ejaculate.

In AI centers, the comfort level of the boar is a major factor which must be satisfied. In our study, a negative correlation between the duration of the semen collection–ejaculation process and boar/dummy temperatures was noticed. When the body and/or dummy temperatures increased, the male felt less comfortable mounting onto the dummy and the ejaculation process was over faster. In the swine industry, the time spent on the semen collection process is included in the overall production cost, because it reflects the technicians’ working hours and efficiency of their productivity. From the practical point of view, the collection of thermal data provides useful information about boar management to improve semen collection efficiency, decrease the overall production cost and reinforce the pig farm’s income. Furthermore, the duration of the semen collection–ejaculation process was positively correlated with OBDA and acceleration. The IMU measures accelerating forces, defines moving changes and sends the real-time signals to the storage device. The results indicated that animals which appeared to move intensely spent more time to conclude the ejaculation process. In that case, a higher volume of ejaculate would be expected. However, no correlation of ODBA and acceleration with semen volume was demonstrated. Perhaps the simultaneously combined negative temperature and positive motion effects affected this parameter. Our study is in agreement with the study by Heinicke et al. [36], who found that the determined accelerometer activity levels were affected by a heat increase. In addition, ODBA was positively correlated with the time-averaged velocity of a sperm head along its average smoothed path, which is the VAP parameter. Earlier studies positively correlated VAP with the number of live-born piglets [27,30]. Thus, forceful and high-frequency movements of a boar towards the three axes of space are associated with higher VAP values and, consequently, with higher possibilities of reproductive efficacy.

Traditionally, salivation during ejaculation has been associated by farmers with vigorous, more active boars, which produce semen of higher quality. This was further supported by a recently published study, which reported that boars with a higher libido showed a higher saliva concentration of oxytocin during and two hours after ejaculation than the day before, indicating that an increase in oxytocin could be associated with positive boars’ emotions during ejaculation [37]. Moreover, boar saliva is usually smeared on the dummy to stimulate the trainee boar to mount and complete the ejaculation. Nevertheless, no correlation was revealed in the present study between salivation and semen characteristics to support this theory. Salivation, however, was associated with ODBA, characterizing the most motile boars, which was positively correlated with the total time of the semen collection process. Therefore, in terms of farm experiences, the intense salivation of the most motile animals was demonstrated, but no association with high-quality sperm was revealed.

## 5. Conclusions

In conclusion, the findings of the present study support the usefulness of the real-time monitoring of boars by body motion sensors and of the body, scrotum and dummy thermal measurements during the semen collection process for the detection of potential sperm quality. These results motivate the further use and development of biomedical tools and techniques for the prognosis of boar fertility based on sperm function and quality traits.

## Figures and Tables

**Table 1:** Correlations that were significant at *p* < 0.05 between sperm variables and thermal data.

Variables		Regression Coefficient	95% Confidence Interval		*p*-Value (*n* = 94)	R-Squared
			Lower Coefficient	Upper Coefficient		
Progressive	Dummy Temperature	−1.298	−1.667	−0.929	<0.001	0.637
Progressive	Scrotal Temperature	−2.064	−2.700	−1.428	<0.001	0.543
Progressive	Body Temperature	−3.697	−4.813	−2.581	<0.001	0.546
Rapid	Dummy Temperature	−1.521	−1.892	−1.151	<0.001	0.662
Rapid	Scrotal Temperature	−2.420	−3.192	−1.647	<0.001	0.576
Rapid	Body Temperature	−4.252	−5.686	−2.818	<0.001	0.577
Slow	Dummy Temperature	1.085	0.736	1.435	<0.001	0.616
Slow	Scrotal Temperature	1.733	1.173	2.294	<0.001	0.516
Slow	Body Temperature	3.262	2.293	4.230	<0.001	0.548
VCL	Dummy Temperature	−0.796	−1.078	−0.514	<0.001	0.625
VCL	Scrotal Temperature	−1.289	−1.862	−0.718	<0.001	0.555
VCL	Body Temperature	−2.378	−3.394	−1.363	<0.001	0.596
VSL	Scrotal Temperature	−0.569	−0.928	−0.209	0.002	0.510
VSL	Body Temperature	−0.911	−1.571	−0.251	0.007	0.515
VSL	Dummy Temperature	−0.369	−0.552	−0.187	<0.001	0.554
ALH	Scrotal Temperature	−0.073	−0.101	−0.044	<0.001	0.518
ALH	Body Temperature	−0.140	−0.190	−0.090	<0.001	0.575
ALH	Dummy Temperature	−0.046	−0.061	−0.030	<0.001	0.602
BCF	Body Temperature	−0.067	−0.121	−0.013	0.016	0.506
BCF	Dummy Temperature	−0.022	−0.037	−0.007	0.005	0.504
Abn. Morph.	Dummy Temperature	0.005	0.004	0.006	<0.001	0.586
Viability	Dummy Temperature	−0.006	−0.007	−0.005	<0.001	0.685
Volume	Scrotal Temperature	−4.630	−8.407	−0.853	0.017	0.756
Volume	Dummy Temperature	−2.560	−4.579	−0.542	0.014	0.760
Tot. Ejac. Time	Dummy Temperature	−0.114	−0.215	−0.014	0.026	0.651

Progressive, Rapid, Slow: spermatozoa with progressive, rapid and slow movement, respectively, %; VCL: curvilinear velocity (μm/s); VSL: straight line velocity (μm/s); ALH: amplitude of lateral head displacement (μm); BCF: beat/cross-frequency (Hz); Abn. Morph.: abnormal morphology %; Volume: ejaculate’s volume (ml); Tot. Ejac. Time: total time of ejaculation process (min). All temperatures were measured in °C.

**Table 2:** Correlations that were significant at *p* < 0.05 between sperm variables, processing of ejaculation and accelerometer sensor data.

Variables		Regression Coefficient	95% Confidence Interval		*p*-Value (*n* = 94)	R-Squared
			Lower Coefficient	Upper Coefficient		
VAP	Acceleration (g)	15.980	1.918	30.042	0.026	0.533
Tot. Ejac. Time	ODBA (g)	26.967	14.009	39.925	<0.001	0.608
Tot. Ejac. Time	Acceleration (g)	40.403	26.606	54.200	<0.001	0.686

VAP: average path velocity (μm/s); Tot. Ejac. Time: total time of ejaculation process (min); ODBA: overall dynamic body acceleration (g); g: acceleration due to gravity (9.81 m/s^2^).

**Table 3:** Correlations that were significant at *p* < 0.05, between salivation, accelerometer sensor and thermal measurements.

Variables		Regression Coefficient	95% Confidence Interval		*p*-Value (*n* = 94)	R-Squared
			Lower Coefficient	Upper Coefficient		
Scrotal Temperature	Body Temperature	1.430	1.211	1.650	<0.001	0.672
Scrotal Temperature	Dummy Temperature	0.445	0.392	0.498	<0.001	0.776
Body Temperature	Dummy Temperature	0.243	0.209	0.277	<0.001	0.767
ODBA (g)	Acceleration (g)	0.969	0.759	1.179	<0.001	0.807
ODBA (g)	Salivation (pixels)	0.008	0.028	0.015	0.005	0.678

ODBA: overall dynamic body acceleration (g); g: acceleration due to gravity (9.81 m/s^2^). All temperatures were measured in °C.

## Data Availability

The data presented in this study are available on request from the corresponding author. The data are not publicly available due to the project’s privancy restrictions.
